# Editorial: Epigenetic mechanisms and post-translational modifications as novel therapeutic targets in cancer

**DOI:** 10.3389/fphar.2022.988334

**Published:** 2022-08-08

**Authors:** Zhi Shi, Ning Wang, Ying-Jie Zhang, Yi-Chao Zheng

**Affiliations:** ^1^ Department of Cell Biology & Institute of Biomedicine, National Engineering Research Center of Genetic Medicine, MOE Key Laboratory of Tumor Molecular Biology, Guangdong Provincial Key Laboratory of Bioengineering Medicine, College of Life Science and Technology, Jinan University, Guangzhou, Guangdong, China; ^2^ School of Chinese Medicine, The University of Hong Kong, Hong Kong, China; ^3^ MOE Key Laboratory of Chemical Biology, Department of Medicinal Chemistry, School of Pharmaceutical Sciences, Cheeloo College of Medicine, Shandong University, Jinan, China; ^4^ Collaborative Innovation Centre of New Drug Research and Safety Evaluation, Zhengzhou, Henan Province, China; ^5^ Key Laboratory of Advanced Drug Preparation Technologies, Zhengzhou University, Zhengzhou, Henan Province, China; ^6^ MOE Key Laboratory of Henan Province for Drug Quality and Evaluation, School of Pharmaceutical Sciences, Zhenghzou University, Zhengzhou, Henan Province, China

**Keywords:** cancer, targets, epigenetic mechanisms, post-translational modifications, methylation

This Research Topic “*Epigenetic mechanisms and post-translational modifications as novel therapeutic targets in cancer*” collected 11 articles on epigenetic mechanisms, post-translational modifications, cell cycle and signaling pathways related to tumorigenesis and development. Our aim is to provide new research directions for targeted cancer therapies and drug development.

Epigenetic modifications affect genetic expression without the sequence change of DNA, and its disorders are closely related to the occurrence and progression of cancer. Methylation is a widely studied form of epigenetic modification. Zhou et al. summarized the role and mechanism of the modification related enzymes “Writers” and “Erasers” in cancer, providing a basis for the development of anti-tumor epigenetic drugs. Tang et al. assessed the causal relationship between smoking-related DNA methylation and breast cancer risk by Mendelian randomization. The results suggest that DNA methylation plays an important role in linking smoking to breast cancer, especially the subtype of ER^+^ breast cancer. Zeng et al. evaluated pyroptosis-related genes in esophageal adenocarcinoma and found that the expressions of GSDMB and ZBP1 were influenced by DNA methylation levels. Huo et al. focused on the role of RNA modification in cancer. They summarized the molecular mechanism of RNA modification in the occurrence and development of head and neck squamous cell carcinoma and discussed the related treatment options. Jing et al. demonstrated that hypoxia induced upregulation of ELFN1-AS1 expression in colorectal cancer cells. As a potential target of competing endogenous RNA, ELFN1-AS1 relieved the inhibition of TRIM14 by sponging miR-191-5p, thus promoting the proliferation and invasion of colorectal cancer cells. Lu et al. elucidated the role of acetylation modification in tumor immunity by focusing on histone acetyltransferases and deacetylases, and discussed the clinical application of acetylation-modified drugs in tumor therapy.

Post-translational modifications increase the protein diversity and play critical roles in regulating the protein activity, localization, and interaction with other molecules. Hou et al. provided evidence that low expression of USP47 in the primary colorectal cancer was associated with disease-free survival. USP47 deubiquitinated and stabilized the expression of transcription elongation factor A3 (TCEA3). Knockdown of UPS47 or TCEA3 can enhance doxorubicin-induced apoptosis and pyrotosis of colorectal cancer cells, which provides a potential target for the treatment of colorectal cancer.

In normal cells, cell cycle and signal transduction are strictly regulated, and their dysregulation and abnormal activation lead to various diseases including cancer. Xia et al. identified aminoquinoline as a novel multi-kinase inhibitor of CDK4/6 and PI3K/AKT for the treatment of hepatocellular carcinoma. Zhao et al. revealed that lysophosphatidic acid induced transactivation of EGFR through MMP-dependent pathway, which upregulated geminin expression and promoted DNA replication in gastric cancer cells. Guo et al. found that a natural microbial product, Ilicicolin A, as a novel EZH2 antagonist, inhibited the EZH2-mediated signaling pathway to enhance the sensitivity of castration-resistant prostate cancer cells to enzalutamide. Yang et al. investigated the role of kinesin family member 2C (KIF2C) in cervical cancer. KIF2C expression was significantly upregulated in cervical cancer, promoted cervical cancer cells proliferation, invasion, and migration. Knockdown of KIF2C inhibited the development of cervical cancer by activating p53 signaling pathway, providing a new target for the treatment of cervical cancer.

In conclusion, the “*Epigenetic mechanisms and post-translational modifications as novel therapeutic targets in cancer*” Research Topic emphasizes that epigenetic and post-translational modification provide new targets for the development of anti-cancer drugs and bring new strategies for treatment of cancer.

